# Establishment of *Neurospora crassa* as a host for heterologous protein production using a human antibody fragment as a model product

**DOI:** 10.1186/s12934-017-0734-5

**Published:** 2017-07-25

**Authors:** David Havlik, Ulrike Brandt, Kathrin Bohle, André Fleißner

**Affiliations:** 1 Division of Pharmaceutical Biotechnology, Fraunhofer Institute for Toxicology and Experimental Medicine (ITEM), Inhoffenstr. 7, Braunschweig, 38124 Germany; 2 Institut für Genetik, Technische Universität Braunschweig, Spielmannstr. 7, 38106 Braunschweig, Germany; 3 Navigo Proteins GmbH, Heinrich-Damerow-Str. 1, 06120 Halle (Saale), Germany

**Keywords:** *Neurospora crassa*, Heterologous proteins, Antibody fragments, Fusion protein, Proteases, Bioreactor

## Abstract

**Background:**

Filamentous fungi are commonly used as production hosts for bulk enzymes in biotechnological applications. Their robust and quick growth combined with their ability to secrete large amounts of protein directly into the culture medium makes fungi appealing organisms for the generation of novel production systems. The red bread mold *Neurospora crassa* has long been established as a model system in basic research. It can be very easily genetically manipulated and a wealth of molecular tools and mutants are available. In addition, *N. crassa* is very fast growing and non-toxic. All of these features point to a high but so far untapped potential of this fungus for biotechnological applications. In this study, we used genetic engineering and bioprocess development in a design-build-test-cycle process to establish *N. crassa* as a production host for heterologous proteins.

**Results:**

The human antibody fragment HT186-D11 was fused to a truncated version of the endogenous enzyme glucoamylase (GLA-1), which served as a carrier protein to achieve secretion into the culture medium. A modular expression cassette was constructed and tested under the control of different promoters. Protease activity was identified as a major limitation of the production strain, and the effects of different mutations causing protease deficiencies were compared. Furthermore, a parallel bioreactor system (1 L) was employed to develop and optimize a production process, including the comparison of different culture media and cultivation parameters. After successful optimization of the production strain and the cultivation conditions an exemplary scale up to a 10 L stirred tank reactor was performed.

**Conclusions:**

The data of this study indicate that *N. crassa* is suited for the production and secretion of heterologous proteins. Controlling expression by the optimized promoter *Pccg1nr* in a fourfold protease deletion strain resulted in the successful secretion of the heterologous product with estimated yields of 3 mg/L of the fusion protein. The fungus could easily be cultivated in bioreactors and a first scale-up was successful. The system holds therefore much potential, warranting further efforts in optimization.

**Electronic supplementary material:**

The online version of this article (doi:10.1186/s12934-017-0734-5) contains supplementary material, which is available to authorized users.

## Background

Filamentous fungi play important roles in numerous biotechnological applications. Examples from a plethora of industrial processes employing fungi include the production of enzymes, organic acids or important secondary metabolites, such as antibiotics or immunosuppressive drugs [1, 2]. The main advantages of fungi as production organisms are their fast growth on a variety of inexpensive media and an enormous metabolic wealth. In addition, filamentous fungi possess an unbeaten secretory capacity, reaching yields of secreted homologous proteins of up to 100 g/L [3]. Because of these many beneficial features, filamentous fungi have also advanced as expression hosts for heterologous proteins. A prominent example is the production of bovine chymosin by *Aspergillus niger* [4–6] yielding about 1 g/L of this heterologous protein. In contrast to prokaryotic microorganisms, fungi also provide eukaryotic posttranslational modifications, which are important for the stability and activity of many secreted proteins.

The filamentous fungus *Neurospora crassa* has long been established as a model organism in basic research covering a wide variety of scientific topics including cell polarity [7], circadian rhythms [8], cell fusion [9] or genome evolution [10]. However, despite a wealth of advantageous features, it has not yet advanced as a host for heterologous protein production in an industrial scale. The benefits of *N. crassa* include a plethora of available genetic and molecular tools, such as a gene knockout mutant collection [11], and exceptional fast and robust growth [12]. For example on synthetic growth media specific growth rates of 0.37 h^−1^ have been reported for *N. crassa* [13] compared to 0.07 h^−1^ in *Trichoderma reesei* [14] or 0.18 h^−1^ in *Aspergillus niger* [15]. *N.* *crassa* is non-toxic, is traditionally used for food production in South East Asia, and is considered to be safe for food and technical applications [16]. In addition, due to its sexual life cycle [17], genetically different strains can be crossed, and mutations, deletions or expression cassettes can very easily be combined into one strain, allowing the development of modular expression systems.

The general capability of *N. crassa* to produce and secrete heterologous proteins has already been demonstrated. Previous studies reported the successful production of a plant protein [18], bovine preprochymosin [19] and RNase A [20], as well as vaccines [21] with yields in the low µg/L up to mg/L range. However, in these studies, analysis was restricted to shake flask cultivations. Large scale approaches (airlift fermenters with volumes up to 6 L) have only been reported for the production of the homologous proteins tyrosinase [22] and acid phosphatase [23]. However, the most prevalent type of bioreactors in biotechnology are stirred tank reactors (STR). A description of growth and development under these culture conditions is therefore a prerequisite for further consideration of this fungus as a production host in industrial applications. To our knowledge, cultivations of *N. crassa* in STR for heterologous protein production have not been reported, yet. So far, cultivations in STR were carried out only in continuous mode of operation for studying the circadian clock [24] or gene expression under different environmental conditions [25].

The main advantage of filamentous fungi as expression systems is their ability to secrete the product into the growth medium. Secretion of heterologous proteins is commonly achieved by their fusion to a highly secreted native protein in a carrier protein approach [26]. A frequently used carrier is glucoamylase, a multi-domain enzyme cleaving starch into glucose monomers. Under inducing conditions, the glucoamylase GLA-1 of *N. crassa* is highly produced and secreted, making up to 17% of all secreted proteins [27]. Glucoamylases consist of an N-terminal catalytic domain and a C-terminal starch binding domain separated by an *O*-glycosylated linker [28]. This structure is especially well-suited as a carrier protein, since by replacing the starch binding domain with the protein of interest the native linker can be employed as a natural connection.

Despite their positive features, filamentous fungi also pose specific challenges in their use as microbial cell factories. The saprophytic fungal life style involves the secretion of catabolic enzymes into the growth environment, including proteases [29]. Once secreted, proteases cleave their substrates into peptides and amino acids, which are taken up by the cell as carbon, nitrogen and sulfur sources. Heterologous protein production therefore requires strict control of protease secretion in order to prevent product degradation. Common approaches of limiting protease activity include genetic modification of the host strain and/or optimization of the culture processes. The former strategy involves for example the inactivation of protease encoding genes or the deletion of central regulators of protease production, such as transcription factors [26, 30, 31]. In the latter approach, cultivation conditions are identified which limit protease activity or production [32–34].

In addition, filamentous fungi adopt various morphologies in liquid cultures, ranging from dispersed hyphae to mycelial pellets or even robust mycelial mats, which accumulate around submerged bioreactor parts [24] thereby hampering the production process. Morphogenesis and productivity are affected by a multitude of culture parameters, including pH value, temperature, aeration rate or media composition [35]. Since no general relationship between morphology and productivity exists, the optimal growth form needs to be determined for every individual production process. A detailed understanding of the environmental factors controlling morphogenesis is therefore a prerequisite for process optimization.

In this study, we set out to optimize *N. crassa* as a host for the production and secretion of heterologous proteins, using the human antibody fragment HT186-D11 [36] (an scFv, single chain fragment variable) as a model product. We identified secreted proteases as a major limitation for using *N. crassa* as a fungal cell factory. To meet this challenge and to improve yields, we combined genetic engineering and bioprocess development, thereby showing that only this combinatorial approach resulted in significant production optimization.

## Results

**Fig. 1:**
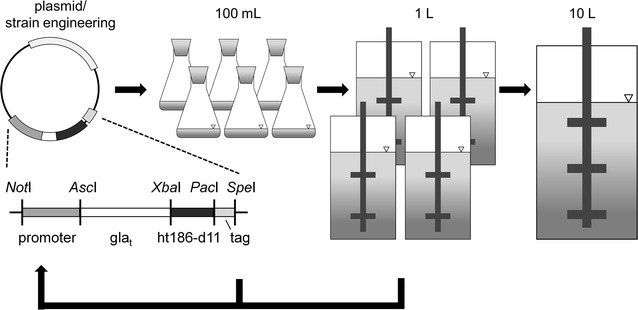
Schematic overview of the production process development. The modular expression cassette comprises a sequence encoding a truncated version of the GLA-1 (*gla*_*t*_), the product gene ht186-d11, and positions for various promoters and tags. Initial tests for optimization of the expression system were conducted in shake flasks cultures. For the subsequent process optimization a parallel bioreactor system was employed. Finally, a first scale-up approach in a 10 L stirred tank reactor was carried out. All optimizations were based on a design-build-test-cycle approach

The aim of this study was to establish *Neurospora crassa* as a production host for heterologous proteins and to analyze and optimize its growth in stirred tank reactors. The work flow of this study is depicted in Fig. 1, ranging from designing an expression system and generating production strains to cultivation in shake flasks (100 mL) and in a parallel bioreactor system (1 L) to finally conducting a first scale-up. Since so far neither robust *Neurospora*-based expression systems nor protocols for cultivation in stirred tank reactors are available, we chose a “design-build-test-cycle” approach, in which the expression cassettes, the host strain, and the growth conditions are optimized in an iterative manner.

### Construction of an expression system

In a first step, a modular expression vector was designed, in which every building block can easily be replaced (Fig. 1). It contains an ht186-d11 human antibody cDNA, which was codon optimized for *N. crassa*. To enable secretion of the heterologous protein, a carrier protein approach was chosen, employing a truncated version of the highly secreted glucoamylase GLA-1 of *N. crassa* as the carrier. GLA-1 homologs are commonly employed to promote secretion of heterologous proteins in filamentous fungi [18, 37, 38]. In the expression vector, the 5′-end of the antibody encoding sequence was fused to the truncated *gla*-*1* sequence (*gla*_*t*_), encoding the catalytic subunit of GLA-1 and the native linker sequence. The starch-binding domain encoding sequence of *gla*-*1* was therefore replaced by the antibody’s ORF. The 3′-end of the antibody gene was fused to a 13×myc-tag [39], for detection by Western blot analysis. To identify a suitable promoter for heterologous expression, we tested three candidate regulatory sequences. First, the *gla*-*1* promoter, which is the starch or maltose inducible native promoter of the GLA-1 carrier encoding gene [40]. Second, the light inducible *vvd* promoter, which allows tunable gene expression [41], and third, the *ccg*-*1* promoter [42, 43], which is routinely used for overexpression in basic research. The three promoters were individually cloned into the expression construct, yielding the plasmids pDH011 (*Pgla*-*1*), pDH014 (*Pvvd*) and pDH016 (*Pccg*-*1*). All constructs were based on plasmid pMF276, which allows targeted, single-copy integration into the *his-3* locus of *N. crassa* [39].

**Fig. 2:**
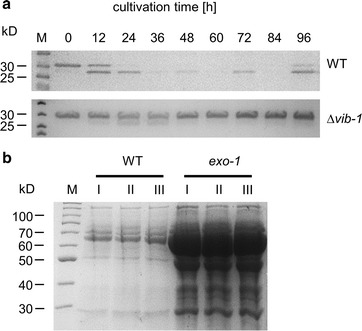
Potential improvement of the production host by deletion of *vib*-*1* and introduction of *exo*-*1*. Deletion of *vib*-*1* results in a significant decrease in protease activity (**a**), and the *exo*-*1* mutation causes hypersecretion of a set of proteins (**b**). To determine protease activity in the culture supernatant (**a**), the wild-type (WT) and the *vib*-*1* deletion strain (*∆vib*-*1*) were cultivated in liquid minimal medium with maltose at 30 °C. Samples of the supernatant were harvested at the indicated time points and incubated for 24 h with 1 µg of HT186-D11. The samples were then analyzed by SDS-PAGE. Presence of the intact antibody fragment is indicated by a band at 30 kD. If protease activity is present in the tested supernatant, smaller degradation products appear or the band fully disappears. To test the hypersecretion of *exo*-*1* strains (**b**), wild-type (WT) and *exo*-*1* were cultivated for 3 days in liquid minimal medium with maltose at 30 °C. Proteins in the supernatant were precipitated, concentrated (factor 30) and separated with SDS-PAGE. *I*–*III* indicates three independent replica. The main band present between 60 and 70 kD represents GLA-1

In choosing a recipient strain for these expression constructs, we considered wild-type protease activity by secreted proteases as one major challenge. Protease activity in the culture supernatant, which can lead to complete degradation of the secreted heterologous product, is a common, major hurdle in establishing filamentous fungi as expression hosts. To test, if production of HT186-D11 would be affected by secreted proteases, the *N. crassa* wild-type strain FGSC #2489 was cultivated in shaking culture for 96 h. Every 12 h, samples of the culture fluid were taken and added to HT186-D11, which had been produced in *E. coli*. After 24 h of incubation, all samples exhibited severe degradation of the antibody fragment, indicating the presence of secreted proteases, which rendered the wild-type strain unsuited for antibody production (Fig. 2a). An earlier study reported that deletion of the *vib*-*1* gene, encoding a transcriptional regulator, results in a strong decrease of protease activity despite starving conditions in *N.* *crassa* [44]. We therefore tested Δ*vib*-*1* (strain FGSC #11309) in the described manner. HT186-D11 was significantly more stable in the presence of Δ*vib*-*1* supernatants, confirming its reduced protease activity (Fig. 2a). We therefore crossed the *vib*-*1* knockout into the *his*-*3* strain (FGSC #9716) to construct the recipient strain DHN-077. Subsequent transformation with pDH011, pDHP014 and pDH016 resulted in the three production strains DHN-117, DHN-118 and DHN-120, respectively.

In the next step, the performance of the three producer strains was analyzed in shake flask cultures. Since the activity of two of the employed promoters is known to be influenced by the carbon source, growth and productivity was compared on minimal medium (MM) containing either sucrose, glucose, acetate or maltose as the sole carbon source. Sucrose is the carbon source of the commonly used Vogel’s MM, while glucose was employed in the initial study describing the use of the *vvd* promoter for tunable expression. Both sugars have however been shown to partially repress *Pccg* *-1* activity, while this inhibition is absent on acetate. Maltose on the other hand is a natural inducer of *gla*-*1*. Cultures were incubated at 15 °C, to promote more precise protein folding at this lower temperature. After 7 days of cultivation the mycelia were harvested and biomass and supernatant were analyzed for the presence of HT186-D11 by Western blot analysis. While in all biomass-associated samples antibodies were detected, only the supernatant of DHN-120 grown on maltose unambiguously contained the antibodies (Additional file 1: Figure S1). While these findings indicate that *N. crassa* is in general able to produce and secrete HT186-D11, the employed production system and growth conditions did not deliver satisfactory results and required further optimization.

Our data indicated that the major amount of produced antibody was biomass associated. However, this analysis could not distinguish if the heterologous protein was not secreted or if it remained linked to the cell wall after secretion. Glucoamylases are commonly associated with the cell wall [28], which could also be the case for our carrier protein construct. Earlier studies in *N. crassa* identified the *exo*-*1* mutant, which significantly hypersecretes GLA-1 into the culture medium, probably because of reduced cell wall association [45, 46]. When we compared general secretion of the wild-type and the *exo*-*1* mutant, this hypersecretion phenotype was confirmed (Fig. 2b). We therefore constructed a second set of production strains by introducing the *exo*-*1* mutation into our original expression isolates. In addition, the *vvd* gene was deleted, since its gene product has been reported to inhibit activity of the *vvd* promoter [41], resulting in strains DHN-177 (*Pgla*-*1*), DHN-201 (*Pvvd*), and DHN-172 (*Pccg*-*1*). The strains were cultivated in modified Vogel’s minimal medium with maltose as the sole carbon source, since in the initial experiment cultivation on this sugar delivered the best results. Since the cultivation at the low temperature of 15 °C had no positive outcome, we cultivated in parallel at 25 °C, which promotes faster and more robust growth of the fungus. Surprisingly, presence of the *exo*-*1* mutation did not improve product levels in the supernatant and only degradation products were detected with DHN-172 (no signals with DHN-177 and DHN-201) (Additional file 1: Figure S2). The *exo*-*1* mutants formed, however, a more homogeneous biomass with less clumps, thereby promoting reproducibility due to easier and more consistent sampling (data not shown). We therefore decided to maintain the *exo*-*1* mutation in the next generation production strains.

We hypothesized that production and secretion of the native glucoamylase might inhibit the production of the carrier-antibody construct in a competitive manner. We therefore introduced a Δ*gla*-*1* mutation into the last tested generation of production strains, resulting in the isolates DHN-178 (*Pgla*-*1*), DHN-182 (P*vvd*) and DHN-176 (P*ccg*-*1*). These strains grew 5–7 times slower than the *gla*-*1* ^+^ isolates when being cultivated in modified Vogel’s minimal medium with maltose, and at least 20% of the sugar was still present at time points when *gla*-*1* ^+^ strains had already depleted the carbon source (data not shown). Full size fusion protein as well as degradation products were detected for the isolates carrying the *Pvvd* and *Pccg*-*1* promoters (Additional file 1: Figure S3). The presence of degradation products indicated that protease activity had reoccurred in these cultures, which was corroborated by protease activity assays using the *E.* *coli*-produced antibody as a substrate (data not shown). Full size fusion protein could probably only be detected because the Δ*gla*-*1* strains had not started to secrete proteases when harvested due to their slower growth. The hypothesized beneficial effects on secretion of the fusion protein were not observed.

Based on these findings, we concluded that the deletion of *gla*-*1* had rather detrimental effects and that optimized production strains therefore carry the Δ*vib*-*1* and *exo*-*1* mutation in a *gla*-*1* ^+^ background and, in case of employing the *vvd* promoter, a *vvd* gene deletion.

### Characterization and optimization of the culture conditions

After establishing the optimized expression systems and production strains, we set out to test the influence of various cultivation parameters. All of the above described experiments were conducted in shaking flask cultures. Since many cultivation parameters, such as dissolved oxygen levels and pH value, can, however, not be controlled in flasks, we turned to a parallel bioreactor system with a working volume of 800 mL for further experiments.

**Fig. 3:**
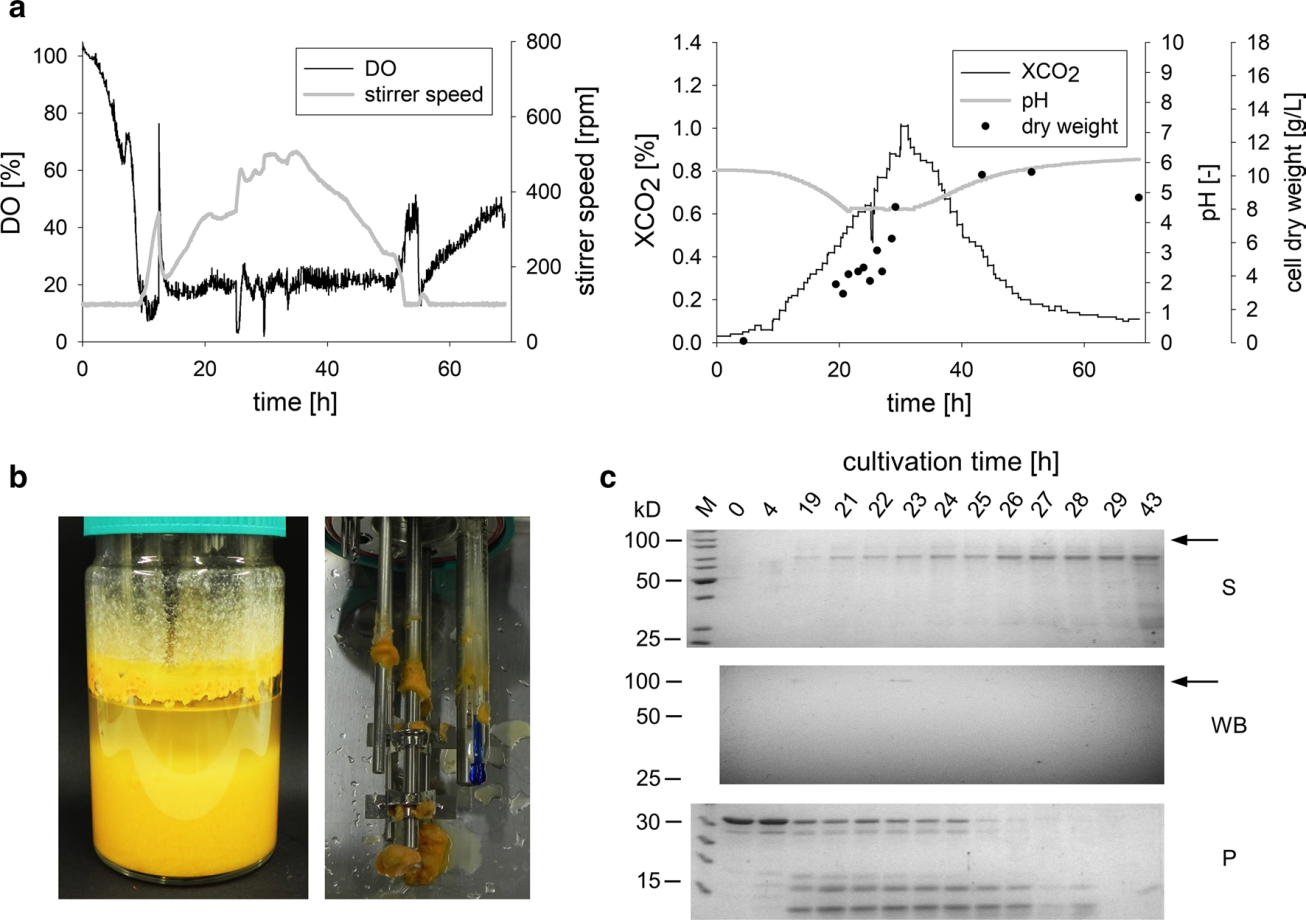
Cultivation of *N.* *crassa* in small scale stirred tank reactors. **a** Growth characteristics of the fungus monitored by online and offline data. Strain DHN-201 (*Pvvd*-*glat*-*ht186*-*13*×*myc, exo*-*1, Δvib*-*1, Δvvd*) was cultivated in a controlled 1 L bioreactor system in minimal Vogel’s medium with maltose as the carbon source at 30 °C. The dissolved oxygen level was maintained at 20% by controlling the stirrer speed. The pH value was maintained above 4.5 by the controlled addition of ammonia. **b** Macroscopical phenotype after 69 h of cultivation. *Left* culture in the bioreactor; *Right* Stirrer after cultivation. **c** Analysis of heterologous fusion protein production. Proteins in the supernatant were concentrated and separated with SDS-PAGE (S). After the Western blot transfer (WB), the tag was detected and signals were developed by electrochemiluminescence (development time of 15 min). The *arrows* indicate the expected molecular mass of the fusion protein. The protease assay (P) was performed as described above

In a first cultivation, strain DHN-201 (*Pvvd*) was grown under constant illumination to induce protein production. The dissolved oxygen level was stably maintained at 20% and the pH value was restricted to a minimum of 4.5, since an earlier study reported maximum hyphal growth on agar at pH 4.5 [47], meaning that it was allowed to rise above but not to fall below 4.5. Since cultivations at low temperatures did not show any advantages, we returned to cultivations at 30 °C which is an established cultivation temperature in basic *N. crassa* research [48–50]. To follow and describe growth of these cultures the parameters dissolved oxygen, stirrer speed, produced CO_2_, pH, and mycelial dry weight were monitored (Fig. 3a). Under these conditions, *N.* *crassa* grew in form of dispersed hyphae and no major hyphal aggregates or pellets were detected. Cultivation of filamentous fungi in stirred tank reactors is frequently hampered by adherence of the biomass at and around the stirring elements. However, the developing biomass did not negatively interfere with the stirring elements of the bioreactor, demonstrating that *N. crassa* is suited for the cultivation in stirred tank reactors (Fig. 3b).

Samples were taken for up to 43 h of cultivation. Proteins were precipitated from the culture filtrate and analyzed by Western blot analysis (Fig. 3c). Only very faint signals for the heterologous protein were detected at time points 19 and 23 h at approximately 105 kD. The protease assay revealed significant protease activity for all time points after 4 h, indicating that the Δ*vib*-*1* mutation does not sufficiently suppress the expression of proteases during cultivation in the stirred tank reactor.

To address this newly arisen protease activity, we turned to optimizing of the culture conditions, focusing first on the amount of nitrogen in the growth medium. To test the influence of the nitrogen source, we substituted the beforehand used Vogel’s minimal medium by Bird medium [51]. Bird medium contains only one type of nitrogen source allowing the systematic analysis of changes in this nutrient concentration. When cultivated on regular Bird medium (containing 25 mM NH_4_^+^), growth of the production strain DHN-201 ceased after 24 h. Interestingly, at this time point the nitrogen source was fully depleted, while only about 50% of the sugar was consumed, suggesting that Bird medium is N-limited. We therefore increased the amount of nitrogen in the medium. As a result, increased NH_4_^+^ concentrations extended the growth period (Additional file 1: Figure S4A–C). However, the desired effect of suppressing the protease activity was not observed (Additional file 1: Figure S4D). For the subsequent experiments, the nitrogen source concentration was, nevertheless, increased from 25 mM ammonium to 65 mM ammonium to prevent further nitrogen limitations. At the same time we changed the nitrogen source from ammonium chloride to ammonium sulfate, to also increase the sulfur concentration, which could also represent a critical factor affecting protease activity.

**Fig. 4:**
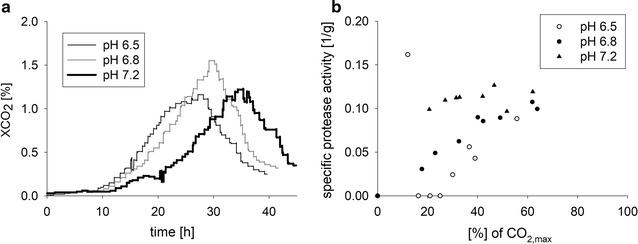
The pH value of the culture medium influences growth and the extracellular protease activity. **a** Growth characteristics and **b** protease activity in cultivations with different pH values. The production strain DHN-201 (*Pvvd*-*glat*-*ht186*-*13*×*myc, exo*-*1, ∆vib*-*1, ∆vvd*) was cultivated in a controlled 1 L bioreactor system in minimal Bird medium with maltose as the sole carbon source at 30 °C. The pH value was controlled at indicated levels by addition of sodium hydroxide throughout cultivation. For the protease activity, the before mentioned protease assay was performed, band intensities were determined via image processing software and ratios calculated in respect to an undigested HT186-D11. Subsequently, those values were divided by the cell dry mass. Due to different growth rates, the culture time had to be normalized. Therefore, the amount of produced CO_2_ at the maximal XCO_2_ value was calculated and defined as 100% (CO_2, max_). Hence, protease activity values at a certain abscissa value are comparable

Protease activity commonly depends on the pH value of the surrounding medium [52–55]. We therefore hypothesized that this product degrading activity could be controlled by pH adjustments. At first, preliminary cultivations with pH values between 4.5 and 9.0 were conducted and the protease activity was determined (data not shown). The three pH values 6.5, 6.8 and 7.2, which still allowed proper growth of the fungus and that were expected to lead to the lowest protease activity, were tested. The protease activity was reduced with decreasing pH values, however, a complete repression of the destructive activity was not achieved (Fig. 4). At pH 6.5, protease activity was significantly delayed compared to the cultures with a higher pH. In addition, the maximum specific protease activity was also reduced at the lowest pH. In summary, these experiments did not fully solve the protease activity issue, however, they revealed optimized ammonium concentrations (65 mM) and an optimal culture pH of 6.5.

**Fig. 5:**
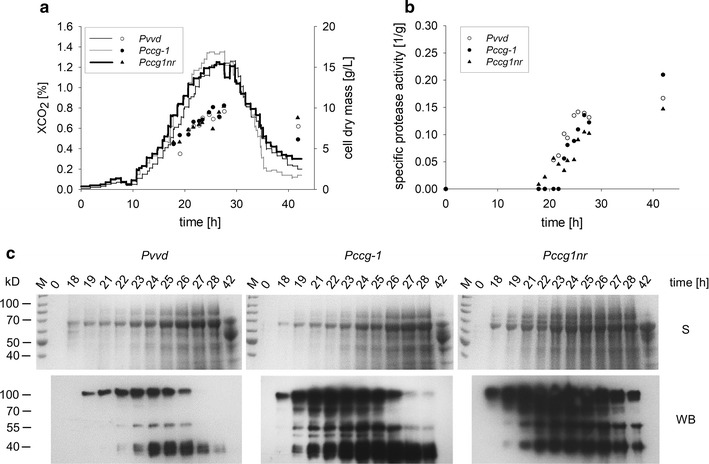
Process optimization results in the production of significant amounts of heterologous protein. **a** Growth characteristics (XCO_2_: graph; dry mass: data points) and **b** protease activity of production strains with different promoters under optimized process conditions. Cultivation was conducted in a controlled 1 L bioreactor system in minimal Bird medium with maltose as the sole carbon source at 30 °C. The pH value was controlled at pH 6.5 by the addition of sodium hydroxide throughout cultivation. Protease activity was determined as stated above. The employed strains were *Pvvd* (DHN-201: *Pvvd*-*glat*-*ht186*-*13*×*myc, exo*-*1, ∆vib*-*1, ∆vvd*), *Pccg*-*1* (DHN-172: *Pccg*-*1*-*glat*-*ht186*-*13*×*myc, exo*-*1, ∆vib*-*1, ∆vvd*) and *Pccg1nr* (DHN-250: *Pccg1nr*-*glat*-*ht186*-*13*×*myc, exo*-*1, ∆vib*-*1, ∆vvd*). **c** Production of the heterologous fusion protein. Concentrated proteins from the supernatant were separated via SDS-PAGE (S) and the product was detected after Western blot transfer (WB). The signal was developed with electrochemiluminescence (development time of 1 s)

To evaluate the impact of the process optimization on the performance of the various production strains carrying the different expression cassettes, cultivations under optimized conditions (constant pH 6.5, 65 mM ammonium) were conducted. Since so far no cultivation condition supported the production of the heterologous fusion protein when the glucoamylase promoter was employed for expressional control, strain DHN-178 was excluded. Instead, *Pccg1nr* was introduced as a new promoter in addition to *Pvvd* and *Pccg*-*1*. This newly employed promoter is a variant of *Pccg*-*1*. Although *Pccg*-*1* is routinely used for overexpressions in *N.* *crassa*, its regulation is complex and it is partially repressed by glucose [42]. An earlier study reported that the deletion of two binding sites of repressor elements in the promoter sequence results in a complete loss of this glucose repression [56]. The production strains DHN-201 (*Pvvd*), DHN-172 (*Pccg-1*), and DHN-250 (*Pccg1nr*) were cultivated in the parallel bioreactor system and production of the heterologous antibody fragment was analyzed (Fig. 5). The choice of promoter did not significantly impact the growth behavior or the protease activity (Fig. 5a, b). Western blot analysis revealed production of the heterologous protein in all three cultivations. Use of the *Pccg1nr* promoter resulted in highest yields, while the *Pvvd* promoter strain produced the least (Fig. 5c). Despite the potential repression of the *Pccg*-*1* promoter by glucose, which is released during metabolism on maltose, product levels were higher with *Pccg*-*1* than with *Pvvd* promoter. In all three cultures, increasing amounts of degradation products were detected in the course of the cultivation leading to a complete loss of all signals after 42 h. Although the protease activity was significantly decreased in the optimized process, the remaining activity still caused complete loss of full size fusion protein indicating that even the combination of the *∆vib*-*1* genetic background with the optimized cultivation conditions is still not sufficient for a satisfactory production process.

### Identification of critical protease genes

Besides mutating a protease regulator gene, such as *∆vib*-*1,* deletion of particular protease genes constitutes another strategy for generating protease deficient isolates [31]. For *N.* *crassa* a gene knockout mutant library is available [11], which allowed the analysis of numerous protease gene deletion mutants. Combining several individual mutations into one production strain is also a simple task, since *N. crassa* is easily sexually crossed under laboratory conditions. We therefore analyzed 26 isolates carrying knockout mutations in predicted protease genes for a reduced ability to degrade the HT186-D11 fragment. Eight of these strains exhibited different degrees of significantly reduced protease activity. To compare the deficiencies of these strains, a score was introduced (Additional file 1: Table S1; Figure S5). The mutations of the four highest scoring strains (*∆spr*-*7, ∆apr*-*9, ∆NCU00263* and *∆apr*-*3,* abbreviated as 4×*∆prot*) were combined into one single production strain. Because *∆apr*-*3* and *∆apr*-*13* mutations had the same score, *∆apr*-*3* was randomly chosen. In addition, the *exo*-*1* mutation was again introduced into the quadruple knockout strain. As an expression cassette the construct carrying the *Pccg1nr* promoter was chosen, which had shown the best performance in the above-described experiments. Since the last cultivations had resulted in secreted heterologous protein, we now also considered purification of this product. Therefore, the 13×myc-tag, which we had used for immunochemical detection of the antibody fragment, was replaced by a his_10_-tag which allows for a more economic method of protein purification. This resulted in a decrease in molecular mass of the fusion protein from 105 to 85 kD. The obtained strain was designated as DHN-270. To compare the effect of the four protease gene deletions to the *∆vib*-*1* deletion, the expression construct was also introduced into a Δ*vib-1*, *exo*-*1* isolate, resulting in strain DHN-252.

**Fig. 6:**
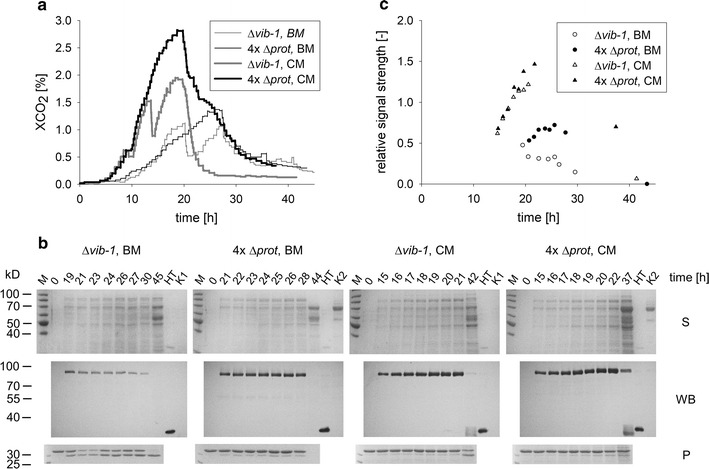
Fourfold protease deletion results in higher protein yields due to lower protease activity. **a** Exhaust gas composition as a representative value for the growth characteristics. Cultivation was conducted in a parallel bioreactor system in minimal Bird medium (BM) and complex medium (CM). The employed strains were *∆vib*-*1* (DHN-252: *Pccg1nr*-*glat*-*ht186*-*10*×*his, exo*-*1, ∆vib*-*1, ∆vvd*) and 4×*∆prot* (DHN-270: *Pccg1nr*-*glat*-*ht186*-*10xhis, exo*-*1, ∆spr*-*7, ∆apr*-*9, ∆apr*-*3, ∆NCU00263*). **b** Analysis of heterologous protein production. Proteins from the supernatant (not concentrated) were separated via SDS-PAGE (S) and the product was detected after Western blot transfer (WB). The signal was visualized directly on the membrane with an NBT/BCIP system. The protease assay (P) was performed as mentioned above. **c** Yield quantification via Western blot analysis. Band intensities in **b** were quantified via image processing software and ratios calculated in relation to HT186-D11 (HT, 25 ng) produced in *E. coli*. As a negative control, strains with an *exo*-*1*, Δ*vib*-*1* background (K1, strain DHN-141) and *exo*-*1* (K2, strain FGSC #2256) were used (no production strains)

Both strains were cultivated in the parallel bioreactor system in optimized Bird medium with maltose as the sole carbon source. In addition, both isolates were also grown on a complex medium consisting of yeast extract, tryptone, malt extract and maltose, since we hypothesized that its constituents, such as peptides and amino acids, could result in a further decrease in protease activity. Since the growth rates of the isolates slightly differed, growth phases were determined by the percentage of the estimated maximal XCO_2_ value (e.g. 80% of XCO_2, max_) observed in preliminary cultivations for better comparability. At comparable stages protease activity and product formation were determined (Fig. 6). As a result, the 4×*∆prot* production strain developed more robustly, while growth of the *∆vib*-*1* strain was characterized by short interruptions that were independent of the growth medium. This growth behavior was not representative for all cultivations but was observed several times in independent experiments (data not shown). Both strains grew faster and reached higher XCO_2_ values in the complex medium (Fig. 6a). In optimized Bird medium, the 4×*∆prot* strain reached significantly higher product levels than the reference strain (protein band at 85 kD). Its protease activity was significantly reduced compared to the Δ*vib*-*1* strain, indicating that the strategy of deleting the protease genes was successful (Fig. 6b, c). Despite the different growth behaviors, both strains produced similar amounts of fusion protein in complex medium, which were higher than in Bird medium. This increased productivity correlated with approximately 40% higher biomass levels (data not shown). For both strains, no significant protease activity was detected in complex medium, supporting our hypothesis on the medium’s influence. Because of the more robust growth and the good performance in defined and complex medium, the 4×*∆prot* production strain (DHN-270) was chosen for further experiments.

### First scale-up approaches

After the optimization of the expression cassette, the genetic background of the recipient strain and the culture conditions resulted in robust production of the HT186-D11 antibody, a first scale-up attempt was conducted.

**Fig. 7:**
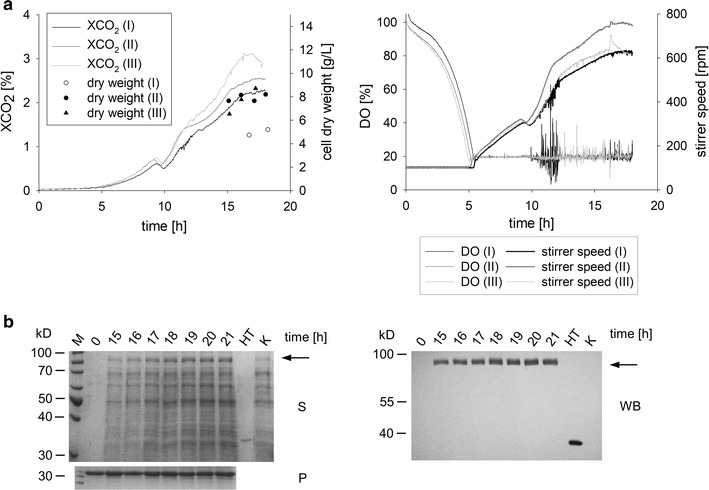
Process scale up to 10 L shows comparable growth and production characteristics. **a** Growth characteristics of three cultivations in 10 L stirred tank reactors (I–III) in complex medium with maltose as carbon source at 30 °C. The employed strain was DHN-270 (*Pccg1nr*-*glat*-*ht186*-*10xhis, exo*-*1, ∆spr*-*7, ∆apr*-*9, ∆apr*-*3, ∆NCU00263*). The dissolved oxygen level and pH value (pH 6.5) were controlled as in the small scale experiments. **b** Production analysis of cultivation II. Proteins from the supernatant were separated via SDS-PAGE (S) and the product was detected after Western blot transfer (WB). As a negative control (K), a comparable production strain missing the his_10_-tag was used (DHN-281, *Pccg1nr*-*glat*-*ht186*-*13xmyc, exo*-*1, ∆spr*-*7, ∆apr*-*9, ∆apr*-*3, ∆NCU00263*). The signal was visualized directly on the membrane with an NBT/BCIP system. The protease assay (P) was performed as mentioned above. As a positive control, HT186-D11 (HT, 10 μg/mL) was used

Strain DHN-270 was grown in complex medium in a 10 L stainless steel stirred tank reactor. Three independent cultivations were conducted to demonstrate reproducibility. The growth behavior was monitored and the production was analyzed by the established methods (Fig. 7). Showing only slight variations, all three cultures grew in a fashion comparable to the development in the parallel bioreactor system. Exhaust gas values as well as dissolved oxygen levels and stirrer speed were similar, indicating that the chosen culture conditions allow for robust and reproducible growth of *N.* *crassa*. No protease activity was observed and the full size product without any degradation products was detected (Fig. 7b). Finally, the product concentration in the culture supernatant was estimated based on the immunostain results by comparing the signal strength of HT186-D11 produced in *E.* *coli* and the signal of the fusion protein after 21 h of cultivation. A total of 3 mg/L of heterologous fusion protein was detected, indicating that the developed expression system allows the production of significant amounts of heterologous protein, therefore providing the basis for further optimizations and novel applications.

## Discussion

The results of this study demonstrate that *Neurospora crassa* is generally suited as a host for heterologous protein production. After the expression system was genetically optimized and the culture conditions were adjusted, a significant amount of heterologous fusion protein was produced. Our study identified secreted proteases as a major roadblock, which we addressed by two different strategies. While deletion of the transcriptional regulator VIB-1 appeared as an efficient method for achieving protease deficiency [44], cultivation in a small laboratory bioreactor demonstrated that the *∆vib*-*1* mutant neither has a sufficiently decreased protease activity nor grows in the robust manner required by industrial production processes. In contrast, the more time consuming second strategy of combining several protease gene deletion mutations resulted in significantly reduced protease activity, while still enabling robust and reproducible growth.

Comparison of the production strains from the two different protease deficiency strategies in defined Bird and complex medium suggested that protease regulation in *∆vib*-*1* strains is more complex than expected. It has long been demonstrated that protease activation is linked to nutrient limitation, including nitrogen, sulphur or carbon starvation [52, 57, 58]. Environmental proteins provide rich sources of these elements and protease secretion is therefore a common part of the fungal starvation response [59]. Consistent with this notion, the addition of different amino acids represses the secretion of certain proteases [60]. This might explain why *∆vib*-*1* production strains did not show any protease activity when being cultivated in complex medium, which contains a mixture of amino acids. In addition, the secreted proteases might use peptides present in complex media as substrates, resulting in an apparent elimination of protease activity in the culture supernatant.

Although the use of complex medium solved the protease activity problem encountered when using the *∆vib*-*1* production strain, this mutation proved unsuited for the construction of expression hosts. In our cultivations, *∆vib*-*1* strains grew in both complex and defined media in an unpredictable manner, indicated for example by unexplained interruptions during exponential growth. It has been reported that VIB-1 is also involved in carbon catabolite repression and in glucose signaling [61]. The deletion of this gene might therefore result in undesirable and difficult to predict metabolic changes during the production process. Taken together these observations prompted us to employ instead the quadruple protease deletion strain for a scale-up.

A key step in optimizing the production process was the adjustment of the media composition, particularly the nitrogen concentration. The original Bird medium possesses a C/N ratio of 24, resulting in a nitrogen limitation [51]. Lowering this ratio to 11 provided the production strain with nitrogen amounts sufficient for extended growth and protein production. When comparing the production media of different filamentous fungi such as *Aspergillus* spp. and *Trichoderma reesei* C/N ratios of 2–6 are described [15, 34, 62, 63], demonstrating further potential for optimization.

Another crucial aspect of our process development was the pH optimization. At pH 6.5 protease activity was mostly suppressed. *N. crassa* secretes a variety of different proteases [64], which possess different pH optima, resulting in their classification as acid [54, 55], neutral [52] and alkaline proteases [53]. Our data indicate that at pH 6.5 most proteases are inactive, suggesting that most of the *N. crassa* proteases secreted under our cultivation conditions are acid proteases.

As part of the genetic optimization strategy, we included the *exo*-*1* mutation into the production strains. The *exo*-*1* mutant was described as a hypersecreting strain overproducing various exoenzymes [45, 46], especially the glucoamylase GLA-1, which we employed as a carrier protein. However, the desired effect of yield improvement was not observed after incorporation of *exo*-*1* into the production strains. The mechanism resulting in hypersecretion in *exo*-*1* is so far not understood. It is possible that the *exo*-*1* effect includes a hyperactivation of the glucoamylase promoter, which in our study was replaced by different promoters. In addition, hypersecretion might require carbon starvation [45, 46], which did not occur in our cultivations. While the introduction of *exo*-*1* into the production strains did not deliver the desired effect, it resulted, however, in more homogeneous mycelial growth in the bioreactor and therefore contributed to reproducible growth and a facilitation of sampling.

Compared to previous reports of heterologous protein production in *N.* *crassa*, our production process resulted in significantly higher amounts of heterologous protein. Considering only the heterologous protein (in our case the scFv of ca. 31 kD) without the carrier, a calculated product concentration of ca. 0.9 mg/L was achieved, while earlier studies reported the production of 10 µg/L of zeamatin [18] or 356 µg/L of RNase A [20]. Only the production of preprochymosin led to a similar concentration of 0.9–1.2 mg/L [19]. Compared to other long-established fungal expression systems, these numbers are still low. In *A. niger* [63] and *T. reesei* [65] yields of up to 100 and 150 mg/L were reached for similar proteins, indicating further optimization potential for *N.* *crassa*.

In other eukaryotic production hosts, such as the yeast *Pichia pastoris,* higher yields between 150 and 300 mg/L were achieved for antibody fragments [66, 67]. The current standard eukaryotic system for the production of pharmaceutical proteins is Chinese hamster ovary cells (CHO). These are usually employed for more complex proteins such as full-length antibodies yielding up to 12 g/L in industrial settings [68].

Although *N.* *crassa* does not achieve such high yields yet, further optimization might be worthwhile due to a number of advantageous features over the common production systems. Due to its long and successful history as a eukaryotic model organism, a plethora of genetic resources and methods are available including the fully sequenced and annotated genome and [64, 69] and a gene knockout mutant library [11]. In addition, targeted integration allows the incorporation of DNA fragments into virtually every genomic locus. Strain construction is therefore reproducible and transcriptionally highly active loci can be targeted for the insertion of expression cassettes. Other potential advantages over CHO cells include the quick development of production host lines by sexual crossing and extremely fast growth, resulting in processing times of a few days rather than several weeks.

Our study also demonstrates that *N.* *crassa* is suited for the cultivation in a stirred tank reactor. Under the chosen conditions the fungus showed free mycelial growth instead of pellet formation, which is the common growth form of many filamentous fungi [70]. Due to the *exo-1* mutation, long filaments did not form in the bioreactor resulting in a homogenous culture broth with a relatively low viscosity (data not shown). The process was easily scaled up from shake flask with a volume of 100 mL medium to 10 L bioreactors. Taking all these advantages into account, *N. crassa* possesses great potential to be further developed into an industrial production host for heterologous proteins.

While the further optimization will involve obvious steps, such as the introduction of a protease cleavage site between carrier and product or the development of a fed-batch process, also more general questions need to be addressed. These include: Why are heterologous proteins not produced and secreted in similar amounts to endogenous proteins? How does codon usage influence productivity? What are the metabolic limitations for the expression of heterologous proteins? In addition, robust and reliable analytics for a product quantification and purification scheme need to be implemented.

## Conclusions

This study describes the successful engineering of the eukaryotic model organism *N.* *crassa* into a production host for heterologous proteins. Key steps included the development of a protease deficient strain, the use of a suited promoter system as well as bioprocess optimization including identification of an optimal pH value and adjustment of the media composition. Only the combination of genetic manipulation and bioprocess optimization allowed the fast and efficient development of a production system for antibody fragments with first yields of almost 1 mg/L, thereby demonstrating the power of interdisciplinary approaches.

## Methods

### Vector construction

Most vectors were constructed by using yeast recombination cloning (YRC), as previously described [11]. After the assembly, a PCR was performed using the outermost primers in order to amplify the desired construct. The antibody fragment gene *ht186*-*d11* was synthesized by GeneArt™ (Thermo Fisher Scientific, Germany) including codon optimization for the expression in *N.* *crassa*. The gene was amplified by PCR and subcloned into respective plasmids. Detailed lists of used and constructed plasmids as well as the primers used are provided in Additional file 1: Tables S2, S3 and S4.

### Construction of *Pccg1nr*

The novel promoter *Pccg1nr* contains two short deletions compared to the original *ccg*-*1* promoter: CRE (*Cyclic AMP Response*, GTGACGTCAC) and NRS (*Neurospora Repressor Site*, TTGCTAGCAA) [56]. As a first step, two PCRs were performed creating DNA strands containing the CRE (primers 920 and 921) or NRS deletion (923 and 925) with the *ccg*-*1* containing plasmid pMF272 as a template. In a second step, yeast recombinational cloning [11] was applied to combine and integrate these two PCR products into the vector pRS426. The deletions were confirmed by sequencing and the *ccg1nr* promoter was amplified for further cloning with the primers 920 and 925.

### *Neurospora crassa* strain construction

Strains used in this study are listed in Additional file 1: Table S5. For the construction of strains via transformation, recipient strains with respective deletions and mutations had to be generated. When combining multiple deletions, crosses were performed in multiple steps on Westergaard’s medium [71]. Mating types were determined by crossing with the *N.* *crassa* wild-types FGSC #2489 and FGSC #988. Strains with single gene deletions were obtained from the *Neurospora* gene knockout library [11]. When combining multiple deletions based on the *hph* cassette, correct strains were identified by PCR analysis using genomic DNA as a template (for primers see Additional file 1: Table S4). Presence of the *exo*-*1* mutation was determined by a plate assay based on starch degradation. Spores of the respective strain were spotted on solid Vogel’s medium containing sorbose (20 g/L) and starch (5 g/L) and incubated for 2–3 days at 30 °C. The agar was then dyed with Lugol’s solution (20 g/L potassium iodide and 10 g/L iodine). Strains carrying the *exo*-*1* mutation developed a clear halo around the spots due to increased starch degradation.

After the construction of recipient strains, transformation of macroconidia with respective plasmids and subsequent screening with PCR for homologous integration and single spore isolation was performed as previously described [72].

### Cultivations in shake flasks

Most cultivations in liquid medium were performed in 250 mL shake flasks with or without baffles containing 100 mL Vogel’s minimal medium with varying carbon sources. The concentration of the carbon sources was 10 g/L in shake flasks, if not indicated otherwise. Standard Vogel’s minimal medium contained 2.5 g/L sodium citrate × 2H_2_O, 5 g/L KH_2_PO_4_, 2 g/L NH_4_NO_3_, 0.2 g/L MgSO_4_ × 7H_2_O, 0.1 CaCl_2_ × 2H_2_O as well as trace elements [73]. To obtain conidia, production strains were grown on solid Vogel’s medium for 7 days in slants or Erlenmeyer flasks. Spores were then harvested and washed three times in deionized water. Cultures were inoculated with a final concentration of 5 × 10^5^ − 1 × 10^6^ spores/mL and incubated at 30 °C and 100 rpm on a rotary shaker (Multitron, Infors HT, Switzerland) or as indicated. Cultures were filter harvested on a Miracloth filter pad (Merck Millipore, USA). The mycelium was rinsed twice with deionized water and either dried at 80 °C for at least 48 h for dry biomass determination or frozen in liquid nitrogen for further analyses.

### Bioreactor cultivations

For the process development, the DASGIP parallel bioreactor system (Eppendorf, Germany) was employed. Cultivations were carried out in four glass bioreactors (SR0700ODLS) including the corresponding gas analyzer and the control software. Cultures were inoculated to a final concentration of 1 × 10^6^ spores/mL. Cultures were grown in Vogel’s minimal medium, Bird medium or complex medium at 30 °C in a working volume of 800 mL. Optimized Bird medium contained 32.5 mM (NH_4_)_2_SO_4_ instead of NH_4_Cl in addition to 20 g/L maltose, 4.85 g/L MES, 1.74 g/L K_2_HPO_4_, 0.174 g/L K_2_SO_4_, 0.058 g/L NaCl, 0.203 g/L MgCl_2_ × 6H_2_O, 0.074 g/L CaCl_2_ × 2H_2_O as well as trace elements [51]. Complex medium was composed of 10 g/L yeast extract, 10 g/L tryptone from casein, 5 g/L malt extract, 4.9 g/L MES and 17 g/L maltose. The aeration was carried out with pressured air and was set to 0.5 vvm. The dissolved oxygen level was maintained at a minimum of 20% by controlling of the stirrer speed with an initial setting of 100 rpm. Used impellers were either two Rushton turbines or pitched blade impellers. The pH was regulated by the addition of 12.5% (v/v) ammonia or 2 M sodium hydroxide (in experiments, in which the influence of the nitrogen concentration was analyzed). If necessary, antifoam agent (Schill + Seilacher “Struktol” GmbH, Germany) was added manually. Dry biomass weight was determined as described above.

Large scale cultivations were carried out in Biostat C stainless steel reactors (Sartorius, Germany). *N.* *crassa* was cultivated in a working volume of 9.0 L with aeration and pH regulation comparable to the laboratory scale. Used impellers were three Rushton turbines and the cultivation was controlled by UBICON software (ESD Electronic System Design, Germany).

### Analysis of protein production

To determine the protein production, the biomass associated fraction and the culture supernatant were analyzed. After cultivation was completed, the biomass was filter harvested (Miracloth filter) and frozen in liquid nitrogen. Protein extraction and sodium dodecylsulphate-polyacrylamide gel electrophoresis (SDS-PAGE) were performed as described previously [74]. Proteins from the culture supernatant were precipitated by adding 0.4 g (NH_4_)_2_SO_4_ per mL of supernatant and incubated for 30 min at 4 °C. Precipitated proteins were pelleted (30 min, 20,000×*g*, 4 °C) and resuspended in 1/30 of the previous culture volume. Protein extracts and precipitated proteins were analyzed by SDS-PAGE including Coomassie Brilliant Blue staining and Western blot analysis. Of crude protein extracts, approximately 50–100 µg of protein were loaded onto the gels, for precipitated proteins 15 µL were loaded. After Western blotting, membranes were blocked with 2% (w/v) milk powder in PBS with 0.1% (v/v) Tween 20 (MPBS-T). The myc-tag was detected by anti c-myc antibody (clone 9E10, 1:5000, Sigma-Aldrich, USA), the his-tag with a 6×His epitope tag antibody (clone HIS.H8, 1:2000 in blocking buffer, Thermo Scientific, USA). Depending on the favored detection sensitivity, the NBT/BCIP system (Nitro blue tetrazolium chloride and 5-Bromo-4-chloro-3-indolyl phosphate, insensitive) or the ECL (electrochemiluminescence, sensitive) was applied.

For the NBT/BCIP system, an anti-mouse IgG (Fc specific) alkaline phosphatase antibody (1:200,000 in blocking buffer, Sigma-Aldrich, USA,) was used as the secondary antibody. Respective protein bands were stained with substrate buffer (0.03% (w/v) NBT, 0.015% (w/v) BCIP, 100 mM Tris HCl, 0.5 mM MgCl_2_, pH adjusted to 9.5). When the ECL system was applied the secondary antibody was F(ab’)2-goat anti-mouse IgG (H + L), HRP conjugate (1:4000 in blocking buffer, Thermo Scientific, USA). The super signal west pico chemiluminescent substrate (Thermo Scientific, USA) was used for signal detection. Exposure times varied between 1 s and 15 min as stated in the figure captions.

### Biochemical analyses

To determine the concentration of maltose and other reducing sugars, a colorimetric assay was performed based on Bernfeld [75]. For the reagents, 12 g of sodium potassium tartrate tetrahydrate were dissolved in 8 mL 2 M NaOH. In parallel, a 96 mM 3,5-dinitrosalicylic acid solution was prepared in 20 mL of purified water. For the final color reagent solution, both solutions were slowly added to 12 mL of deionized water. All solutions were prepared at 60 °C. The solution was stored in a dark environment. The concentration of reducing sugars was determined by adding 200 µL of the sample to 100 µL of the color reagent solution and heating to 100 °C for 15 min. After cooling to room temperature, 900 µL of deionized water were added and the absorption at a wavelength of 540 nm was measured. Final concentrations were calculated by comparing the values to a standard calibration.

The ammonium concentration was determined by using a commercial kit based on a UV method (R-Biopharm, Germany). The measurement was conducted according to the manufacturer.

### Protease assay

As substrates for the protease activity assays, different scFv including HT186-D11 were produced in *E.* *coli* using the expression vector pOPE101-XP. Production, isolation and purification of the scFv was performed as previously described [76]. The protease assay was conducted by incubating filtrated supernatants (Miracloth filter) with 1 µg of purified HT186-D11 followed by SDS-PAGE analysis. Missing bands of the scFv at 30 kD indicated protease activity specific for the given scFv. For the comparison of protease activity between different samples, the intensities of the respective protein bands were determined with the image processing software ImageJ (https://imagej.nih.gov/ij/) and ratios were calculated in respect to an undigested scFv.

## Additional file

**Additional file 1.** Additional figures and tables.

## Abbreviations

BA: biomass associated
BCIP: 5-Bromo-4-chloro-3-indolyl phosphate
CHO: Chinese hamster ovary (cells)
CRE: Cyclic AMP Response
EC: extracellular
ECL: electrochemiluminescence
FGSC: Fungal Genetic Stock Center
*gla*-*1*: glucoamylase 1
HRP: horseradish peroxidase
MM: minimal medium
MPBS-T: PBS with milk powder and Tween 20
NBT: nitro blue tetrazolium chloride
NRS: Neurospora Repressor Site
ORF: open reading frame
PBS: phosphate buffered saline
P*ccg*-*1*: clock controlled gene promoter
P*gla*-*1*: glucoamylase promoter
P*vvd*: vivid promoter
scFv: single chain fragment variable
SDS-PAGE: sodium dodecylsulphate-polyacrylamide gel electrophoresis
STR: stirred tank reactor
*vib*-*1*: vegetative incompatibility blocked
XCO_2_: percentage of CO_2_ in exhaust gas
