# Supplementary material for: Establishment of Neurospora crassa as a host for heterologous protein production using a human antibody fragment as a model product
Source: Microb Cell Fact. 2017 Jul 25;16:128. doi: 10.1186/s12934-017-0734-5 (PMC5526295; doi:10.1186/s12934-017-0734-5)
Supplement: Supplementary file 1 — Additional file 1. Additional figures and tables. [file 12934_2017_734_MOESM1_ESM.docx]

Figure S1: The use of different promoters yields different amounts of produced heterologous fusion protein. Cultures were grown at 15 °C in shake flasks containing 100 mL minimal Vogel’s medium with 18 g/L glucose, 20 g/L sucrose, 12 g/L acetate and 10 g/L maltose. (A) The biomass-associated (BA) and (B) extracellular (EC) fractions were analyzed after 7 days of incubation. The biomass was disrupted, associated proteins isolated and 1 µg of total protein was separated. Proteins from the supernatant were concentrated. Both fractions were analyzed via Western blot analysis, detecting the *myc*-tag. Strains and promoters were the following: (a) glucoamylase promoter (DHN-117: *Pgla-glat-ht186-13×myc, ∆vib-1*), (b) *vvd* promoter (DHN-118: *Pvvd-glat-ht186-13×myc, ∆vib-1*) and (c) *ccg-1* promoter (DHN-120: *his-3+::Pccg-1-glat-ht186-13×myc, ∆vib-1*). As controls, (d) a strain with a tagged version of the truncated glucoamylase (DHN-129: *Pgla-glat-13×myc, ∆vib-1*), (e) a strain with a full version of GLA-1 and integration at the native locus (DHN-075: *gla-1::gla-1-ht186-d11, ∆vib-1*) as well as (f) a negative control (*∆vib-1*) were cultivated. The arrow without asterisk indicates the expected molecular mass of the fusion protein, the arrow with asterisk indicates the tagged GLA_t_. The signals in the Western blot analysis were visualized directly on the membrane with an NBT/BCIP system.

Figure S2: *exo-1* incorporation into *gla-1*^+^ production strains does not improve product yields in the supernatant. Cultivations were performed in shake flasks in 100 mL minimal Vogel’s medium at 15 °C for 90 h and at 25 °C for 42 h. (A) The biomass associated (BA) and (B) extracellular (EC) fractions were analyzed. The biomass was disrupted, associated proteins isolated and 1 µg of total protein was separated. Proteins from the supernatant were concentrated. Both fractions were analyzed via Western blot analysis detecting the *myc*-tag. The expression cassette comprised the codon optimized *ht186-d11* gene and (a) the glucoamylase promoter (DHN-177: *Pgla-glat-ht186-13×myc*), (b) the *vvd* promoter (DHN-201: *Pvvd-glat-ht186-13×myc*) and (c) the *ccg-1* promoter (DHN‑172: *Pccg-1-glat-ht186-13×myc*). Additionally, (d) a strain with codon non-optimized expression construct (DHN‑210: *Pccg-1-glat-ht186orig-13×myc*) and as a control (e) a strain without the expression cassette (DHN-169: *his-3+*) were analyzed. All strains had a *∆vib-1, ∆vvd, exo-1* background. Further controls were (f) a production strain without *exo-1* background (DHN-120: *Pccg-1-glat-ht186-13×myc, ∆vib-1*) and as a negative control (g) DHN‑141 (*∆vib‑1, exo-1*). Signals in the Western blot analysis were developed by electrochemiluminescence (development time 2 min).

Figure S3: The heterologous fusion protein is produced by *exo-1* *∆gla-1* strains but degradation occurs. Cultivation were performed in shake flasks in 100 mL minimal Vogel’s medium at 15 °C for 120 h and at 25 °C for 57 h. (A) The biomass associated (BA) and (B) extracellular (EC) fraction were analyzed. The biomass was disrupted, associated proteins isolated and 1 µg of total protein was separated. Proteins from the supernatant were concentrated. Both fractions were analyzed via Western blot analysis detecting the *myc*-tag. The expression cassette of the production strains comprised the codon optimized *ht186-d11* gene and (a) the glucoamylase promoter (DHN-178: *Pgla-glat-ht186-13×myc*), (b) the *vvd* promoter (DHN-182: *Pvvd-glat-ht186-13×myc*) and (c) the *ccg-1* promoter (DHN‑176: *Pccg-1-glat-ht186-13×myc*). Additionally, (d) a strain with codon non-optimized expression construct (DHN‑211: *Pccg-1-glat-ht186orig-13×myc*) and as a control (e) a strain without the expression cassette (DHN-170: *his-3+*) were cultivated. All strains had a *∆vib-1, ∆vvd, ∆gla-1, exo-1* background. Signals in the Western blot analysis were developed by electrochemiluminescence (development time 2 min).


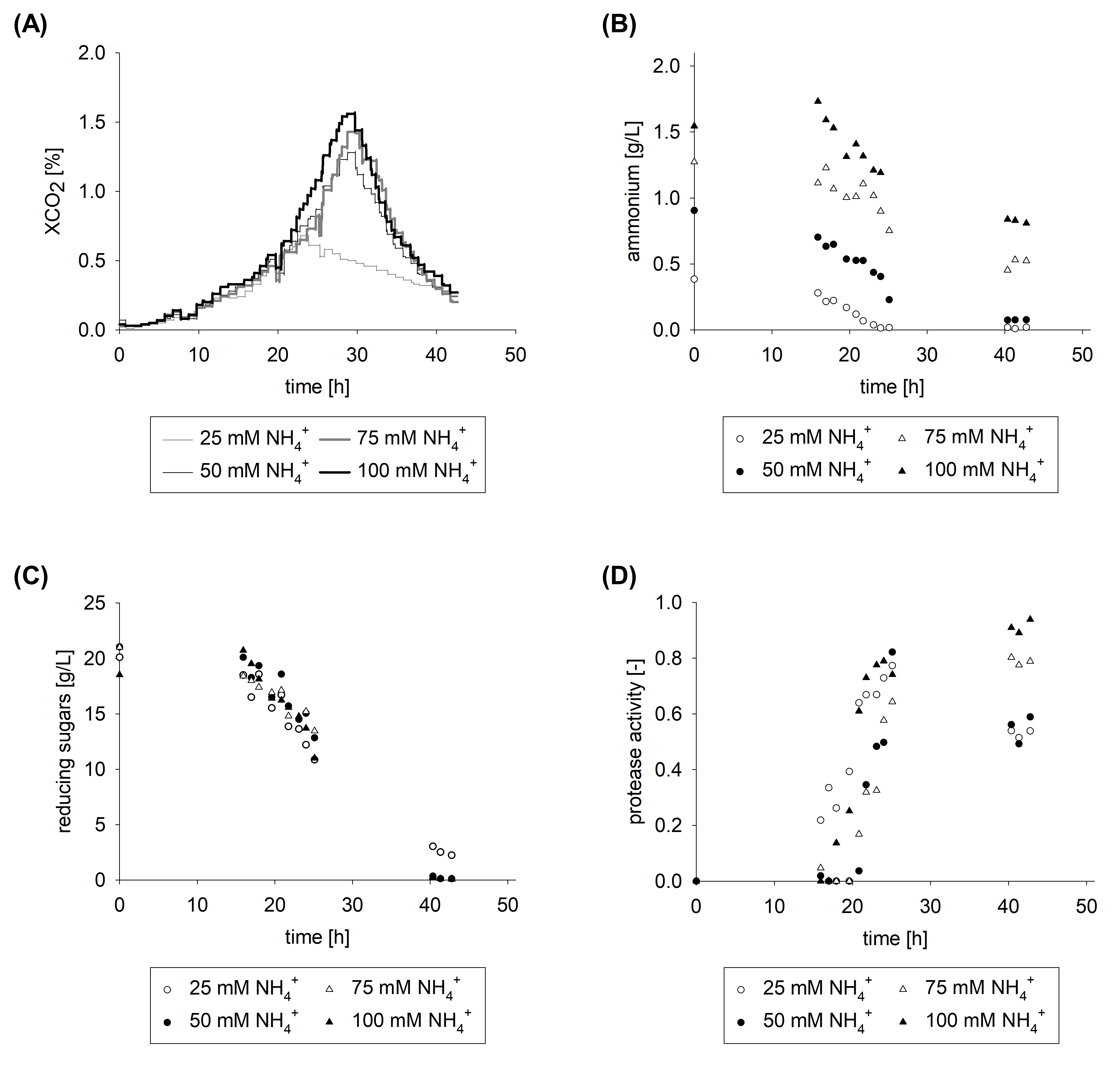


Figure S4: Bird medium’s carbon/nitrogen ratio is suboptimal. The production strain DHN-201 (*Pvvd-glat-ht186-13×myc, exo-1, ∆vib-1, ∆vvd*) was cultivated in a controlled 1 L bioreactor system in minimal Bird medium with maltose as the sole carbon source at 30 °C and varying concentrations of ammonium. The dissolved oxygen level was maintained at 20 % by controlling the stirrer speed. The pH value was kept stable by the controlled addition of sodium hydroxide with a set minimal value at pH 4.5. (A) Exhaust gas composition; displayed as a representative data set for the online data. (B-D) Development of different growth parameters, which were biochemically determined offline: (B) Ammonium, (C) Reducing sugars and (D) Protease activity. Protease activity was determined via the before mentioned protease assay. Band intensities were determined via image processing software and ratios calculated by normalizing to an undigested HT186-D11.

Figure S5: Determination of the protease activity score. Single deletion mutants from the knock-out library were cultivated multiple times for 40 h in 100 mL minimal Vogel’s medium and the supernatant used in a protease assay. The protease assay was interpreted by comparing the pattern of degradation with the wild-type pattern. Wild-type degradation was rated with 0 points, complete absence of protease activity with 4 points. D, H, S and T are abbreviations for the different antibody fragments used in the assay (D1.3, HT186‑D11, SH511-B1 and TOB5-D4).

Table S1: Protease deletion mutants tested in this study. Single deletion mutants from the knock-out library were cultivated multiple times for 40 h in 100 mL minimal Vogel’s medium and the culture supernatants were used in a protease assay. The protease assay was interpreted by comparing the pattern of degradation with the wild-type pattern. Wild-type degradation was rated with 0 points, complete absence of protease activity with 4 points. The average value is displayed.

| **Locus** | **Gene name** | **Encoded protein** | **Score** |
| --- | --- | --- | --- |
|  |  |  |  |
| NCU07159 | spr-7 | proteinase T | 3.0 |
| NCU07533 | apr-9 | secreted aspartic proteinase | 1.7 |
| NCU00263 | - | serin endopeptidase | 1.5 |
| NCU02059 | apr-3 | endothiapepsin | 0.7 |
| NCU10907 | apr-13 | PEPad | 0.7 |
| NCU00994 | apr-2 | endothiapepsin | 0.5 |
| NCU03168 | apr-5 | aspartic-type endopeptidase | 0.5 |
| NCU09484 | apr-12 | hypothetical protein | 0.5 |
| NCU00338 | apr-1 | aspartic proteinase | 0 |
| NCU00673 | spr-4 | serine protease p2 | 0 |
| NCU00831 | - | extracellular serine carboxypeptidase | 0 |
| NCU01151 | mpr-1 | calpain-B | 0 |
| NCU03219 | spr-3 | serine protease-3/Kex2 | 0 |
| NCU03355 | mpr-2 | metalloprotease-2/calpain-5 | 0 |
| NCU04269 | apr-6 | aspartyl proteinase | 0 |
| NCU04430 | - | leupeptin-inactivating enzyme 1 | 0 |
| NCU04903 | - | tripeptidyl peptidase I | 0 |
| NCU04953 | apr-7 | penicillopepsin | 0 |
| NCU05980 | - | carboxypeptidase S1 | 0 |
| NCU06055 | spr-5 | extracellular alkaline protease | 0 |
| NCU06720 | - | carboxypeptidase cpdS | 0 |
| NCU06834 | - | ADAM protease ADM-B | 0 |
| NCU07063 | apr-8 | aspartyl protease-8/hypothetical protein | 0 |
| NCU08739 | apr-10 | endothiapepsin | 0 |
| NCU09350 | - | aspartic-type endopeptidase | 0 |
| NCU09992 | - | serine peptidase | 0 |

Table S2: List of plasmids used in this study.

| **Plasmid** | **Vector** | **Insert** | **Description** | **Source** |
| --- | --- | --- | --- | --- |
|  |  |  |  |  |
| pDH001 | pOPE101-XP | ht186-d11 | expression vector for *E. coli* | Hust *et al*., 2007 |
| pDH006 | pRS426 | gla5'-ht186-Tgla-hph-gla3' | assembled expression cassette for integration at *gla-1* locus | this study |
| pDH011 | pMF276 | Pgla-glat-ht186-13xmyc | integration at *his-3* locus | this study |
| pDH013 | pMF276 | Pgla-glat-13xmyc | integration at *his-3* locus | this study |
| pDH014 | pMF276 | Pvvd-glat-ht186-13xmyc | integration at *his-3* locus | this study |
| pDH016 | pMF276 | Pccg-1-glat-ht186-13xmyc | integration at *his-3* locus | this study |
| pDH017 | pMF276 | Pccg-1-glat-htorig-13xmyc | integration at *his-3* locus | this study |
| pDH020 | pMF276 | Pccg1nr-glat-ht186-13xmyc | integration at *his-3* locus | this study |
| pDH021 | pMF276 | Pccg1nr-glat-ht186-10xhis | integration at *his-3* locus | this study |
| pCSN44 | - | - | contains hph cassette | FGSC |
| pRS426 | - | - | Cloning vector for Yeast Recombinational Cloning | FGSC |
| pMF276 | - | - | see reference | Honda & Selker, 2009 |

Table S3: Details of vector construction.

| **Plasmid** | **Cloning procedure** |
| --- | --- |
|  |  |
| pDH-011 | YRC, PCR with primers 694/796, restriction with *Not*I and *Pac*I and ligation into pMF276 |
| pDH-013 | PCR with primers 694/802 and genomic DNA (wild-type) , restriction with *Not*I and *Pac*I and ligation into pMF276 |
| pDH-014 | YRC, PCR with primers 797/796, restriction with *Not*I and *Pac*I and ligation into pMF276 |
| pDH-016 | PCR with primers 831/832 and plasmid pMF272, restriction with *Not*I and *Asc*I and ligation into pDH-014 |
| pDH-017 | PCR with primers 909/910 and plasmid pDH-001, restriction with *Xba*I and *Pac*I and ligation into pDH-016 |
| pDH-020 | YRC, PCR with primers 920/925, restriction with *Not*I and *Asc*I and ligation into pDH-016 |
| pDH-021 | YRC, PCR with primers 920/925, restriction with *Not*I and *Asc*I and ligation into a pMF276 based plasmid with Pccg1-glat-10xhis |

Table S4: List of primers used in this study.

| **Primer-No.** | **Name** | **Sequence** |
| --- | --- | --- |
|  |  |  |
| 12 | bem1_center_for | AAGAATGGAGCCATGGTTTATGGT |
| 13 | bem1_center_rev | TTCAAGAGGGAACTCGGTTAGGA |
| 21 | his3-f | CTTGCAGTCTTGCACGTTG |
| 22 | his3-r | CTCTCGAGTCCCGTTATTGC |
| 82 | HPH F | GTCGGAGACAGAAGATGATATTGAAGGAGC |
| 83 | HPH R | GTTGGAGATTTCAGTAACGTTAAGTGGAT |
| 317 | hph-test-r | TCGTCCGAGGGCAAAGGAATAGAG |
| 684 | gla5'-NotI-Yeast-for | GTAACGCCAGGGTTTTCCCAGTCACGACGGCGGCCGCTCCACAACTTCGACCC CGCTG |
| 685 | gla5'-rev | CCTCCACGTATCATCAAGAGTCG |
| 686 | D1-3-Linker_gla-for | CTCTTGATGATACGTGGAGGGGCGGCGGCGGCTCCGGCGGCGGCGGCTCCGGC GGCGGCGGCTCCGAAGTCAAGTTGCAAGAATC |
| 687 | D1-3-Term_gla-rev | CCTAGTACGAAGCAAGCGATTCAGTGGTGATGATGATGATGGGACAAATC |
| 688 | HT186-D11-Linker_gla-for | CTCTTGATGATACGTGGAGGGGCGGCGGCGGCTCCGGCGGCGGCGGCTCCGGC GGCGGCGGCTCCCAGATGCAGCTCGTCCAGTC |
| 689 | HT186-D11-Term_gla-rev | CCTAGTACGAAGCAAGCGATTCAGTGGTGATGGTGGTGGTGGGAGAGGTC |
| 690 | Tgla-for | ATCGCTTGCTTCGTACTAGG |
| 691 | Tgla-hph-rev | ATATCATCTTCTGTCTCCGACCAAGCATATATACCACGG |
| 692 | gla3'-hph-for | ACGTTACTGAAATCTCCAACGTCAAAACAAAACCCTAAAA |
| 694 | Pgla5'-NotI-Yeast-for | GTAACGCCAGGGTTTTCCCAGTCACGACGGCGGCCGCCCACTCATTTCCTTCA CCAT |
| 707 | Tgla-SpeI-Yeast-neu-rev | GCGGATAACAATTTCACACAGGAAACAGCACTAGTCAAGCATATATACCACG GCA |
| 708 | gla3'-NotI-Yeast-neu-rev | GCGGATAACAATTTCACACAGGAAACAGCGCGGCCGCAGGGCGCCGAGCAGA AGGAG |
| 727 | vib1-test F | CTCTGATGGCCTGGGTTATTT |
| 731 | pRS426-seq-f | TTCAGGCTGCGCAACTGTTG |
| 732 | gla5'-seq-f | CCAGCACCACCGTCACCCCT |
| 733 | HT186-seq-f | TCGGCTCCAAGTCCGTCCAC |
| 734 | D1.3-seq-f | AACAAAAGCAAGGTAAATCC |
| 735 | Tgla-seq-f | GCTTCTCAACAAAATTTCAA |
| 736 | hph1-seq-f | ATGCAGCTCTCGGAGGGCGA |
| 737 | hph2-seq-f | GCCGATAGTGGAAACCGACG |
| 738 | gla3'-seq-f | TCTTGAGACCCTCGTAGAGG |
| 739 | Pgla-seq-f | GACCAGGGTTCACCAGCCGG |
| 740 | gla-seq-f | CTGGCTTCGATCTCTGGGAG |
| 741 | spr-7-test-f | ATCAGTTTTGTAGGCGTGCC |
| 742 | apr-9-test-f | AGTAGTTTTGGTGTAGGCGT |
| 743 | apr-3-test-f | AGGGGGACGACTTTGGGGAG |
| 744 | NCU00263-test-f | CATTTAGTGGCTTTGGACGG |
| 745 | Pvvd-NotI-f | ACTACTGCGGCCGCGCAGTGGCATCAAACACAAGC |
| 755 | gla5-neu-seq | GCTCTGGTCCACCACTATTG |
| 756 | gla3-test-r | TTCCCAGATCTTCCAGATGC |
| 767 | Pgla-test-f | CCACTCATTTCCTTCAC |
| 768 | Pgla2-seq-f | TTCATGGTCGACCTCCAG |
| 776 | gla5-extra-seq-f | GTCCGCCAACTCTCTGCCC |
| 794 | glat-XbaI-HT186-r | ACTGGACGAGCTGCATCTGTCTAGAGCCGGTCTGCGTGGGAGGGGTGAC |
| 795 | HT186-XbaI-f | AGACCGGCTCTAGACAGATGCAGCTCGTCCAGTCCGAGG |
| 796 | HT186-PacI-Yeast-r | GCGGATAACAATTTCACACAGGAAACAGCGTTAATTAAGGAGGACGGGAAGA GGGTGACG |
| 797 | Pvvd-NotI-Yeast-f | GTAACGCCAGGGTTTTCCCAGTCACGACGGCGGCCGCGCAGTGGCATCAAACA CAAGCCG |
| 798 | Pvvd-AscI-glat-r | AGCGAAGAGACGAGATGCATGGCGCGCCGGTGCTGGTTATGAGACAGTG |
| 799 | glat-AscI-Pvvd-f | AGCACCGGCGCGCCATGCATCTCGTCTCTTCGCTCC |
| 802 | glat-PacI-r | AGTAGTGTTAATTAAGCCGGTCTGCGTGGGAGGGGTGACGGTGGTGC |
| 803 | HT186-test-f | AAGAAGCCTGGCGCCTCCG |
| 804 | HT186-test-r | TTGGGCTGGCCGAGGACGG |
| 808 | vvd-test-f | AACCTATACGGTGCAAGTTGTCG |
| 809 | Pgla3-seq-f | TCAAGACCGTCCTCTCGG |
| 810 | Pvvd1-seq-f | TATCCTGCTGGTGATCATCCC |
| 811 | Pvvd2-seq-f | TTAAGGTGTCAAGTGTCG |
| 812 | Pvvd3-seq-f | AAGTGTGAAGCATATATGGC |
| 813 | Pvvd4-seq-f | TCTCCATACAGGACCCTGGG |
| 814 | gla175-seq-f | TTCTGTGCAACATCGGTGC |
| 831 | Pccg1-NotI-f | ACTACTGCGGCCGCTAGAAGGAGCAGTCCATCTGC |
| 832 | Pccg1-AscI-r | TGATGAGGCGCGCCGATTTGGTTGATGTGAGGGG |
| 833 | pMF276-test-r | TGCAGCCCGGGGGATC |
| 863 | gla-1-test-f | TTCCATCTACAGCGCGAGGG |
| 909 | HT186_orig-XbaI-f | ACTACTTCTAGACAGATGCAGCTGGTACAGTCTGAGGC |
| 910 | HT186_orig-PacI-r | AGTAGTTTAATTAAAGACGACGGGAACAGAGTGACCG |
| 920 | Yeast-NotI-pccg1-f | GTAACGCCAGGGTTTTCCCAGTCACGACGGCGGCCGCTAGAAGGAGCCCTCTCCC |
| 921 | pccg1-credel-r | AAGTGAGGCAATCTTCGTCCTTCACCCGTTGTCTGCCCCTTTGAACCCCTCTCCC |
| 923 | pccg1-nrsdel-f | AAGGACGAAGATTGCCTCACTTCTTTTGCCTGCAAAGAAGGCGC |
| 925 | pccg1-AscI-Yeast-r | GCGGATAACAATTTCACACAGGAAACAGCGGCGCGCCGATTTGGTTGATGTGAGGGG |
| 928 | HTorig-seq | AAAGACGGCCAGGATTACC |

Table S5: *N. crassa* strains used in this study

| **Strain** | **Genotype** | **Vector** | **Recipient strain** | **Parental strain** | **Source** |
| --- | --- | --- | --- | --- | --- |
|  |  |  |  |  |  |
| FGSC #2489 | *mat A* (wild-type) | **-** | **-** | **-** | FGSC |
| FGSC #988 | *mat a* (wild-type) | **-** | **-** | **-** | FGSC |
| FGSC #6103 | *his-3^-^, mat A* | **-** | **-** | **-** | FGSC |
| FGSC #9716 | *his-3^-^, mat a* | **-** | **-** | **-** | FGSC |
| FGSC #9718 | *∆mus-51::bar^+^, mat a* | **-** | **-** | **-** | FGSC |
| FGSC #13284 | *∆apr-3::hph, mat a* | - | - | **-** | FGSC |
| FGSC #13334 | *∆apr-7::hph, mat A* | - | - | **-** | FGSC |
| FGSC #14728 | *∆apr-13::hph, mat a* | - | - | **-** | FGSC |
| FGSC #13553 | *∆spr-5::hph, mat a* | - | - | **-** | FGSC |
| FGSC #14776 | *∆apr-9::hph, mat a* | - | - | **-** | FGSC |
| FGSC #11309 | *∆vib-1::hph, mat A* | - | - | **-** | FGSC |
| FGSC #11869 | *∆NCU00263::hph, mat A* | - | - | **-** | FGSC |
| FGSC #11847 | *∆mpr-1::hph, mat a* | - | - | **-** | FGSC |
| FGSC #11951 | *∆apr-10::hph, mat A* | - | - | **-** | FGSC |
| FGSC #12071 | *∆mpr-2::hph, mat a* | - | - | **-** | FGSC |
| FGSC #12115 | *∆spr-7::hph, mat A* | - | - | **-** | FGSC |
| FGSC #12334 | *∆spr-4::hph, heterokaryon* | - | - | **-** | FGSC |
| FGSC #12323 | *∆NCU06720::hph, mat A* | - | - | **-** | FGSC |
| FGSC #16097 | *∆NCU09350::hph, mat A* | - | - | - | FGSC |
| FGSC #13518 | *∆NCU04903::hph, mat A* | - | - | - | FGSC |
| FGSC #13726 | *∆NCU05980::hph, mat A* | - | - | - | FGSC |
| FGSC #16399 | *∆NCU00831::hph, mat a* | - | - | - | FGSC |
| FGSC #16457 | *∆NCU04430::hph, mat A* | - | - | - | FGSC |
| FGSC #16538 | *∆apr-2::hph, mat a* | - | - | - | FGSC |
| FGSC #14075 | *∆apr-5::hph, mat a* | - | - | - | FGSC |
| FGSC #14719 | *∆apr-8::hph, mat A* | - | - | - | FGSC |
| FGSC #14721 | *∆apr-12::hph, mat A* | - | - | - | FGSC |
| FGSC #14847 | *∆NCU06834::hph, mat A* | - | - | - | FGSC |
| FGSC #17477 | *∆apr-1::hph, mat A* | - | - | - | FGSC |
| FGSC #18064 | *∆NCU09992::hph, mat A* | - | - | - | FGSC |
| FGSC #20115 | *∆spr-3::hph, mat a, heterokaryon* | - | - | - | FGSC |
| FGSC #18891 | *∆apr-6::hph, mat A* | - | - | - | FGSC |
| DHN-049 | *∆spr-7::hph, ∆apr-9::hph, mat a* | - | - | FGSC #12115 x  FGSC #14776 | this study |
| DHN-054 | *ΔNCU00263::hph, Δapr-3::hph, mat A* | - | - | FGSC #11869 x  FGSC #13284 | this study |
| DHN-063 | *gla-1::gla-1-ht186-d11-Tgla-hph, ∆mus-51::bar^+^, mat a* | cassette from pDH006 | FGSC #9718 | - | this study |
| FGSC #11556 | *∆vvd::hph, mat A* | - | - | - | FGSC |
| DHN-075 | *gla-1::gla-1-ht186-d11-Tgla-hph, ∆vib-1::hph, mat A* | - | - | DHN-063  x  FGSC #11309 | this study |
| DHN-077 | *his-3^-^, ∆vib-1::hph, mat a* | - | - | FGSC #11309 x  FGSC #9716 | this study |
| FGSC #2256 | *exo-1, mat a* | - | - | - | FGSC |
| DHN-084 | *his-3^-^, ∆vib-1::hph, ∆vvd::hph, mat a* | - | - | FGSC #11556 x  DHN-077 | this study |
| FGSC #7825 | *∆gla-1::hph, mat A* | - | - |  | FGSC |
| DHN-117 | *his-3^+^::Pgla-glat-ht186-d11-13xmyc,  ∆vib-1::hph, mat a* | pDH011 | DHN-077 | - | this study |
| DHN-118 | *his-3^+^::Pvvd-glat-ht186-d11-13xmyc,  ∆vib-1::hph, mat a* | pDH014 | DHN-077 | - | this study |
| DHN-120 | *his-3^+^::Pccg-1-glat-ht186-d11-13xmyc,  ∆vib-1::hph, mat a* | pDH016 | DHN-077 | - | this study |
| DHN-129 | *his-3^+^::Pgla-glat-13xmyc, ∆vib‑1::hph, mat a* | pDH013 | DHN-077 | - | this study |
| DHN-137 | *his-3^-^, exo-1, mat a* | - | - | FGSC #2256  x  FGSC #6103 | this study |
| DHN-141 | *∆vib-1::hph, exo-1, mat a* | - | - | DHN-075  x  FGSC #2256 | this study |
| DHN-148 | *∆gla-1::hph, ∆vib-1::hph, ∆vvd::hph, mat A* | - | - | FGSC #7825  x  DHN-084 | this study |
| DHN-157 | *his-3^-^, ∆vib-1::hph, ∆vvd::hph, exo-1, mat a* | - | - | DHN-148 x DHN-137 | this study |
| DHN-160 | *his-3^-^, ∆vib-1::hph, ∆vvd::hph, ∆gla-1::hph,  exo-1, mat a* | - | - | DHN-148 x DHN-137 | this study |
| DHN-169 | *his-3^+^,∆vib-1::hph, ∆vvd::hph, exo-1, mat a* | - | DHN-157 | - | this study |
| DHN-170 | *his-3^+^, ∆vib-1::hph, ∆vvd::hph, ∆gla-1::hph,  exo-1, mat a* | - | DHN-160 | - | this study |
| DHN-172 | *his-3^+^::Pccg-1-glat-ht186-d11-13xmyc,  ∆vib-1::hph, ∆vvd::hph, exo-1, mat a* | pDH016 | DHN-157 | - | this study |
| DHN-176 | *his-3^+^::Pccg-1-glat-ht186-d11-13xmyc, ∆vib‑1::hph, ∆vvd::hph, ∆gla-1::hph, exo-1,mat a* | pDH016 | DHN-160 | - | this study |
| DHN-177 | *his-3^+^::Pgla-glat-ht186-d11-13xmyc,  ∆vib-1::hph, ∆vvd::hph, exo-1, mat a* | pDH011 | DHN-157 | - | this study |
| DHN-178 | *his-3^+^::Pgla-glat-ht186-d11-13xmyc,  ∆vib-1::hph, ∆vvd::hph, ∆gla-1::hph, exo-1,mat a* | pDH011 | DHN-160 | - | this study |
| DHN-182 | *his-3^+^::Pvvd-glat-ht186-d11-13xmyc, ∆vib-1::hph, ∆vvd::hph, ∆gla-1::hph, exo-1,mat a* | pDH014 | DHN-160 | - | this study |
| DHN-201 | *his-3^+^::Pvvd-glat-ht186-d11-13xmyc,  ∆vib-1::hph, ∆vvd::hph, exo-1, mat a* | pDH014 | DHN-157 | - | this study |
| DHN-210 | *his-3^+^::Pccg-1-glat-htorig-13xmyc, ∆vib-1::hph, ∆vvd::hph, exo-1, mat a* | pDH017 | DHN-157 | - | this study |
| DHN-211 | *his-3^+^::Pccg-1-glat-htorig-13xmyc, ∆vib-1::hph, ∆vvd::hph, ∆gla-1::hph, exo-1, mat a* | pDH017 | DHN-160 | - | this study |
| DHN-224 | *his-3^-^, ∆apr-3::hph, exo-1, mat a* | - | - | DHN-054 x DHN-137 | this study |
| DHN-228 | *∆apr-3::hph, ∆apr-9::hph, ∆spr-7::hph, ∆NCU00263::hph, mat A* | - | - | DHN-049 x DHN-054 | this study |
| DHN-231 | *his-3^-^, ∆apr-3::hph, ∆apr-9::hph, ∆spr-7::hph, ∆NCU00263::hph, exo-1, mat a* | - | - | DHN-228 x DHN-224 | this study |
| DHN-250 | *his-3+::Pccg1nr-glat-ht186-d11-13xmyc, Δvib-1::hph, Δvvd::hph, exo-1, mat a* | pDH020 | DHN-157 | - | this study |
| DHN-252 | *his-3^+^::Pccg1nr-glat-ht186-d11-10xhis,  ∆vib-1::hph, ∆vvd::hph, exo-1, mat a* | pDH021 | DHN-157 | - | this study |
| DHN-270 | *his-3^+^::Pccg1nr-glat-ht186-d11-10xhis,  ∆apr-3::hph, ∆apr-9::hph, ∆spr-7::hph, ∆NCU00263::hph, exo-1, mat a* | pDH021 | DHN-231 | - | this study |
| DHN-281 | *his-3^+^::Pccg1nr-glat-ht186-d11-13xmyc,  ∆apr-3::hph, ∆apr-9::hph, ∆spr-7::hph, ∆NCU00263::hph, exo-1, mat a* | pDH020 | DHN-231 | - | this study |
